# Delineating the Common Biological Pathways Perturbed by ASD’s Genetic Etiology: Lessons from Network-Based Studies

**DOI:** 10.3390/ijms18040828

**Published:** 2017-04-14

**Authors:** Oded Oron, Evan Elliott

**Affiliations:** Molecular and Behavioral Neurosciences Lab, Bar-Ilan University, Faculty of Medicine, 13215 Safed, Israel; odedoron@gmail.com

**Keywords:** ASD, autism, networks, genetics, Fragile-X Syndrome, Wnt, mTOR, Calmodulin, Calcium, NGF

## Abstract

In recent decades it has become clear that Autism Spectrum Disorder (ASD) possesses a diverse and heterogeneous genetic etiology. Aberrations in hundreds of genes have been associated with ASD so far, which include both rare and common variations. While one may expect that these genes converge on specific common molecular pathways, which drive the development of the core ASD characteristics, the task of elucidating these common molecular pathways has been proven to be challenging. Several studies have combined genetic analysis with bioinformatical techniques to uncover molecular mechanisms that are specifically targeted by autism-associated genetic aberrations. Recently, several analysis have suggested that particular signaling mechanisms, including the Wnt and Ca^2+^/Calmodulin-signaling pathways are often targeted by autism-associated mutations. In this review, we discuss several studies that determine specific molecular pathways affected by autism-associated mutations, and then discuss more in-depth into the biological roles of a few of these pathways, and how they may be involved in the development of ASD. Considering that these pathways may be targeted by specific pharmacological intervention, they may prove to be important therapeutic targets for the treatment of ASD.

## 1. The Genetic Basis of Autism

Autism Spectrum Disorder (ASD) is a developmental disorder characterized by persistent deficits in social communication, as well as restricted and repetitive patterns of behavior. It has been well characterized that genetic aberrations have a prominent role in the etiology of ASD [1]. Among the first studies to support a genetic etiology were twin studies published in the 1970s by Folstein and Rutter [2,3]. A recent meta-analysis by Rutter, encompassing a total of 6413 twins, showed that the heritability rate in families with an autistic proband was in the range of 64–91% [4]. Furthermore, it has been well-established that ASD overlaps at both the behavioral and genetic levels with other disorders such as social anxiety, Attention Deficit and Hyperactivity Disorder (ADHD), Intellectual Disability (ID), bipolar disorder and schizophrenia [5,6,7,8]. For example, Khazanda et al. discovered 23 genes, many of which are involved in circadian entrainment, that are associated with ASD, bipolar disorder and schizophrenia, therefore demonstrating shared genes and genetic pathways between various psychiatric disorders [7]. As such, common behavioral phenotypes of these disorders may have a root in shared genetic etiology. 

A heterogeneous genetic etiology for ASD has been firmly established. Historically, some of the first genes that were successfully associated to autism were those responsible for syndromic forms of autism. Thus far, approximately 35 such syndromes have been documented [9]. A sub-group of these syndromic autisms are mendelian monogenic, and consistently develop due to rare mutations. For example, in the case of Fragile-X Syndrome (FXS), which develops due to aberrations in the Fragile-X Mental Retardation 1 (*FMR1*) gene, up to 30% of these male patients also develop ASD [10]. Cortical Dysplasia-focal Epilepsy (CDFE) syndrome is caused by mutations in Contactin Associated Protein 2 (*CNTNAP2*), where a subgroup of patients express autistic behavior [11]. Tuberous sclerosis is caused by mutations in either Tuberous Sclerosis 1/2 (*TSC1*/*TCS2*), where up to 61% of patients express autistic behaviors [12]. Rett syndrome, which includes autistic behavior, is caused by mutations in the Methyl CpG binding protein 2 (*MECP2*) [13]. Another group of syndromic autisms arise from Copy Number Variations (CNV) [14], which contain large duplication/deletion loci that usually encompass several genes, yet the causative gene is often not known. One such example is Phelan-McDermid syndrome where up to 9 Mb are deleted at Chr22q13 encompassing up to 130 genes [15]. The most notable gene in this CNV is *SHANK3*/*PROSAP2*, where mutations in this gene alone have been shown to be associated with ASD [16,17]. Additional CNVs include the 15q11-13 deletion or duplication syndromes [18], the chromosome 16p11.2 deletion or duplication syndromes [19,20] and the 2q23.1 microdeletion syndrome [7]. Most syndromic autisms, such as CDFE, TSC or the 2q23.1 microdeletion syndrome are considered extremely rare, however the highest discovery rate observed in FXS accounts for no more than 2% of autistic cases [9].

A wider search for ASD’s genetic etiology started with linkage studies, which uncovered several candidate loci, including 2q, 3q 7q and 20p. However, it was primarily through Next Generation Sequencing (NGS) technologies that majority of genes were discovered, and by 2012, 10–20% of ASD cases had a known genetic link [9]. One approach for locating new genetic candidates involved in ASD development is to determine de-novo mutations in gene coding regions, usually found in sporadic familial cases. In 2011, O’Roak et al. conducted the first exome-wide sequencing study in ASD on 20 sporadic cases, revealing de-novo mutations in *FOXP1*, *GRIN2B*, *SCN1A* and *LAMC3* [21]. In 2012, in an effort to discover de-novo mutations, the Simons Simplex Collection conducted three large exome-sequencing trails which included approximately 750 families with affected and unaffected siblings. Many promising genes were identified, including *SCN2A*, *CHD8* and *NTNG1* [22,23,24]. By that time, it had become increasingly clear that genetic mutations involved in ASD do not fall into one particular biological category, but seem to be found in genes involved in several different biological systems. In recent years, whole genome sequencing studies are also beginning to appear, which aim to discover genetic aberrations in both coding and noncoding regions. One such study, by Yuen et al., found genetic aberrations in *STXBP1*, *UBE3A*, *KATNAL2*, *THRA*, *KCNQ4*, *MYH14*, *GJB6* and *COL11A1*, some of which have been previously associated with ASD and some with overlapping conditions such as hearing loss [25]. In addition, several Genome-Wide Association Studies (GWAS) emphasized the significance of common genetic variation to the inheritance of autistic traits [26]. 

To this day, over 800 genes have been identified and associated with ASD development [27]. Given this heterogeneous genetic reality, it has been hypothesized that the multitude of genetic aberrations are affecting specific molecular pathways that might be in common across the autistic spectrum, and are responsible for dysregulated neurodevelopment [28]. Discovering such common pathways has proven to be challenging. To address this difficulty, it is appropriate to perform network-based analysis of autism associated genes [29]. In the following chapters, we will discuss attempts at using network-based approaches to discover candidate signaling pathways that are effected by ASD-associated genetic aberrations, and how these signaling pathways may be involved in dysregulated neurodevelopment and the phenotypes of ASD.

## 2. Searching for Common Molecular Targets of Autism Mutations

### 2.1. Protein–Protein Network Analysis and Pathways Enrichment Tools

One approach to discovering molecular pathways that are commonly targeted by autism-associated mutations is to perform Protein-Protein Interaction (PPI) network analysis to identify groups of proteins within a given list of proteins that physically interact with each other. This analysis uses different databases that curate experimentally validated or predicted protein-protein interactions [29]. PPI also identifies hubs, highly interconnected proteins, which might prove to be central in the molecular pathway. However, this approach has some disadvantages and biases. First, tissue specific PPI databases are scarce, which are necessary to understand protein interactions in specific tissues, such as the brain. A very partial solution to this problem is to combine PPI databases with RNA expression from the specific tissue, therefore making sure that you only take into account proteins which are found in that specific tissue. Additional tools used to reveal biologically-relevant pathways within a gene list are gene ontology databases and signaling pathway enrichment tools. The Gene Ontology project initiated by the Gene Ontology Consortium (GOC) strives to provide a unifying vocabulary to genes and their biological roles [30]. In addition, there is a growing number of signaling pathway enrichment tools available for free or through commercial license [31]. There are several differences that should be considered when using these tools, such as method of data curation (manually and computerized), algorithms used to generate signaling pathway maps, types of pathway maps (e.g., disease specific, kinase signaling, hormone mediated) and breadth of knowledgebase. For example, in the next section, we mention studies which used KEGG and IPA pathway enrichment tools to generate pathways that might be affected in ASD. Due to the fact that IPA is a commercial tool, it is difficult to thoroughly compare it to other tools, and thus, reviews conducting such comparisons lack information about IPA. However, one important difference between KEGG and IPA is the size of their knowledgebase. KEGG mainly curates interaction and reactions of molecular pathways from the literature, while IPA also imports data from several external databases, which increases its knowledgebase substantially [32,33]. Therefore, it is necessary to integrate knowledge from several different pathway analysis tools in order to discover biological pathways that are commonly targeted by autism-associated mutations.

### 2.2. Autism-Associated Signaling Pathways Discovered Using Network Analysis and Pathway Enrichment Tools

As part of the effort conducted by the SSC in 2012, O’Roak et al. sequenced the exome of 209 families with sporadic cases of ASD, revealing 126 genes which had truncation or missense mutations, followed by PPI analysis, to determine if these genes form a biologically-relevant network [23]. They discovered one highly interconnected cluster of mutated genes, which included genes involved both in chromatin-binding and β-catenin function. An additional network analysis conducted with the IPA tool enriched 8 Wnt-signaling regulators, which are involved in the regulation of β-catenin, as we will explain later. Therefore, combination of exome sequencing with downstream network analysis revealed an autism-associated molecular pathway, the Wnt-β-catenin pathway. This was not the first study to suggest the involvement of Wnt-signaling in ASD [34,35], however it strongly demonstrated that network analysis may reveal pathways that are highly important for further research. In a 2014 follow up bioinformatics study which included the combined data for the O’Roak et al. study, and two other de-novo ASD mutation studies that were published in the same issue of *Nature*, a subset of these de-novo mutations were enriched for genes involved in chromatin-binding [36]. A separate study collected genome association data of rare CNVs from 6742 ASD patients, and constructed PPI networks based on the genes deleted or duplicated due to the CNVs. Calmodulin1, which is a major regulator of Calcium-signaling, was found as a central hub in the PPI network [37]. An additional network analysis of autism-associated genes has suggested a role for both Wnt and calcium pathways in ASD. Wen et al. performed KEGG pathway analysis on the SFARI annotated list of autism-associated genes. They found that these genes were particularly enriched for biological pathways including Calcium-signaling, Wnt-signaling, mTOR, and cellular adhesion molecules [38]. 

A different strategy to identify molecular pathways that are dysregulated by autism-associated mutations is to use publicly available data of gene expression patterns in the human brain to build gene expression networks and PPI networks that are found in specific brain regions, and then to probe if any of these networks are enriched for autism-associated genes, and are therefore likely to be perturbed by autism-related mutations. Lin et al. used such an approach to identify PPI networks in the brain that are effected by the autism-associated CNV at chromosome 16p11.2 [39]. Using published databases of gene expression and protein interaction data, the authors built protein interaction networks that appear in specific brain regions, and at specific developmental time points. The authors then discovered that four of these protein interaction networks would be disrupted by the CNV, particularly during mid-fetal stage and in early childhood. Gene ontology analysis of these protein networks showed enrichment for Wnt-signaling and NGF-signaling. A similar method was used to discover human brain protein networks dysregulated by rare or common variants associated with autism. Ben-David et al., built brain-region specific gene co-expression modules using WGCNA [40]. They found that a co-expression module that was highly effected by both rare and common autism-associated variants was enriched for genes involved in synaptic transmission and the Calmodulin-binding pathway. To summarize, these studies used different methodologies to uncover molecular pathways targeted by ASD mutations and implicated the Wnt-signaling and Ca^2+^/Calmodulin-signaling [37,38,39,40]. Therefore, we will focus on defining these two specific pathways and how they may be related to the autism phenotype, followed by a short discussion of some of the other pathways identified.

## 3. Wnt-Signaling

### 3.1. Roles for Wnt-Signaling in Neurodevelopment and Adult Brain

Wnt-signaling pathways orchestrate cellular proliferation, polarity and differentiation; processes that are crucial for healthy tissue morphogenesis, especially in the embryonic stage [41]. Dysregulation of these pathways have been implicated in a wide variety of cancers, as well as in other pathologies, such as type II diabetes, osteoporosis and heart conditions [41,42]. In humans there are 19 Wnt glycoprotein ligands, which activate signal transduction pathways through binding to one of the 10 Frizzled receptors. As of today, there are two extensively researched Wnt pathways; a canonical pathway and a non-canonical pathway. In the canonical pathway the secreted Wnt glycoproteins bind to Frizzled receptors, as well as either the LRP6 or LRP5 co-receptors, to initiate a signaling cascade. The activated receptor recruits the scaffolding protein Dishevelled, which recruits and sequesters the Axin-GSK3β-APC destruction complex, thus preventing its role in phosphorylating β-catenin, leaving it stable for downstream activity. Stabilized β-catenin is transported to the nucleus where it fulfills its role as a co-transcription factor by binding to the TCF/LEF transcription factors, or it relocates to the cellular membrane where it maintains cell-cell adhesion complexes [41,42]. The two non-canonical Wnt pathways are independent of β-catenin, and include the Wnt/JNK pathway, which drives polarized cell movements such as neuronal crest migration, as well as the Wnt/Calcium pathway that has been shown to be involved in cardiac development [43]. 

Canonical Wnt-signaling has a pivotal role both in the developing and mature brain. Much evidence has indicated that during development the Wnt pathway regulates the balance between proliferation and differentiation of neuronal progenitor and precursor cells, as was recently reviewed by Noelanders and Vleminckx [44]. In transgenic mice with overexpression of stabilized β-catenin, neuronal precursor cells in the ventricular zone continue to divide beyond their natural timeframe, resulting in enlarged brains and deeper sulci folds that resemble higher mammals [45]. Concurrently, inhibition of β-catenin during embryonic development leads to premature differentiation of precursors to neurons [46]. These studies suggest that Wnt-signaling promotes proliferation of neuronal precursors and delays their differentiation. There is some evidence suggesting Wnt-signaling may inhibit proliferation in certain circumstances. For example, as shown in developing zebrafish, induced activation of Wnt8 reduced the population of neuronal progenitor in the hypothalamus; reduction in neuronal progenitors was also induced by the canonical Wnt pathway activator, BIO [47]. These studies suggest that Wnt-signaling can regulate neuronal precursor proliferation in a manner that is either species-specific, or cell type-specific. 

In addition to its roles in development, Wnt-signaling also affects Neuronal Stem Cell (NSC) proliferation and differentiation in the mature brain. One study of Wnt-signaling in NSCs used mice which express lacZ under the control of a TCF transcription response [48]. These mice received intraventricular infusions of the mitotic inhibitor Ara-C, which kills the dividing progenitor cells and decreases NSCs; followed with BrdU infusions, which allows the tracking of the regeneration of NSCs in the Subventricular Zone (SVZ). 30% of the BrdU positive cells displayed active Wnt-signaling (LacZ); and when Wnt-signaling was pharmacologically blocked, the number of neurospheres in the SVZ significantly decreased. Wnt-signaling has also been shown to induce NSC proliferation after injury [48]. Mice subjected to a stroke-inducing surgery (Pial Vessel Disruption, or PVD) displayed an increase in lacZ expressing neuronal stem cells of the SVZ after seven days. Other studies showed that Wnt-signaling is also involved in the differentiation of NSCs in the mature brain. For example, in adult NSCs of the SVZ, Wnt-signaling induces cell-cycle exit in the presence of Hipk1, an interactor of β-catenin. Furthermore, when overexpressing β-catenin and Hipk1, an increase of the cell-cycle inhibitor P16Ink4 was observed [49]. Therefore, Wnt has a role both in the proliferation and differentiation of NSCs cells in the adult brain.

Apart from its role in NSCs, Wnt-signaling has a positive developmental role in the maturation of dendrites and spines. The α-catenin/β-catenin/*N*-cadherin complex acts as a scaffold between intracellular actin and extracellular cadherin-dependent interactions [50,51]. This role is important for dendrite arborization in the developing brain, as demonstrated by the fact that overexpression of β-catenin, α-catenin and *N*-cadherin individually, and co-expression of β-catenin and *N*-cadherin in vitro, both induce increased dendritic branching [52]. In addition, β-catenin has also been implicated in spine pruning and maturation as part of the β-catenin/*N*-cadherin complex. By inducing a conditional knockout of β-catenin in the cerebral cortex and hippocampus of mice, spine density is increased and is comprised mostly from immature spines [53]. This suggests that pruning of immature spines may be dependent on β-catenin function. 

In addition to the roles in neuronal differentiation and morphology, β-catenin has been suggested to have an additional role in neurotransmission through two distinct mechanisms. First, translocation of β-catenin to spines may increase the size of the post synaptic density (PSD) which leads to increased synaptic transmission [54]. This was shown by inducing a point mutation, which prevents the phosphorylation of Tyr654 in β-catenin, and results in its increased localization to spines. Considering the role of β-catenin in the α-catenin/β-catenin/*N*-cadherin complex, and the complex’s importance in inducing cell-cell contacts [55], the authors deciphered if increased presence of β-catenin in spines might increase synaptic contacts, and affect other properties such as postsynaptic morphology and neurotransmission. Interestingly, they observed more intense and larger PSD-95 puncta and increased mEPSCs. Second, in the presynaptic neuron, β-catenin has been shown to have a role in recruiting vesicles to the presynaptic membrane: β-catenin has a PDZ domain used to recruit PDZ domain containing proteins such as Veli and Cadherin clusters [56]. Veli has been shown to create a tripartite complex with CASK and Mint1, which binds Munc18-1. This complex is essential for vesicle docking to the plasma membrane [57,58]. It has also been shown that regulation of synaptic vesicle release into the synaptic cleft is important for healthy development of synaptic plasticity in 4–8 day old rats [59]. In that regard, β-catenin is suggested to have an essential role. This has been shown by siRNA-mediated knockdown of synaptic β-catenin, which increased spillage of vesicle content into the synaptic cleft in nascent presynaptic terminals [60]. An additional study revealed that neuronal cultures treated with Wnt8A-conditioned media displayed increased PSD95 puncta, and that the co-receptor LRP6 selectively localizes to excitatory synapses where it takes part in regulating excitatory synaptic development [61]. These studies show that Wnt-signaling, and in particular β-catenin, is essential for normal brain development as well as adult brain performance.

### 3.2. How Wnt-Signaling May Be Involved in ASD

From the known roles of Wnt-signaling in neuronal differentiation, morphology, and neurotransmission, we can propose possible roles for dysregulated Wnt-signaling in the processes that are dysregulated in the brain of individuals diagnosed with ASD. Numerous studies have witnessed differences in cortical patterning or spine morphology in the brain of individuals with ASD. In some cases, it has been shown that cortical dendritic spine density is increased in the brain of individuals with autism [62]. This matches the phenotype of the β-catenin knockout mice, which displays increased spines [53]. However, it is not clear if the increase in spine density in the ASD brain is due to an increase in immature spines, as is in the case of the knockout mice. In addition, studies have shown dysregulation of cytoarchitecture in the cortex and in the architecture of the microcolumns in the cortex of individuals diagnosed with ASD [63,64]. A separate study, using the three dimensional imaging technique CLARITY, determined abnormal connections between axons in the brain of individuals with ASD [65]. Considering that dysregulation of Wnt-signaling affects development of normal cortical architecture, as well as neuronal morphology, it is possible that dysregulated Wnt-signaling is involved in these dysregulations in the ASD brain. 

Experimentation in mouse models have also given more insight into the possible roles of Wnt in the development of ASD. Interestingly, a conditional knockdown of β-catenin in Parvalbumin (PV) neurons in mice induced increased repetitive behaviors and anxiety, decreased social interaction, and an increase in PV neuron density in the prefrontal cortex [66]. An additional Wnt-signaling component, Glycogen Synthase Kinase-3 (GSK3) was recently reviewed as a key driver of ASD development, as well as a therapeutic target for FXS [67,68]. GSK3 is hyperactive in several brain regions of the FXS mouse model [69] and impaired social preference and exaggerated anxiety during social interaction have been recorded in a GSK3 knockin mouse model [35]. So using this model, researchers inhibited GSK3 functioning in the hippocampus to explore how its hyperactivity contributes to FXS phenotypes. Both novel object detection and hippocampal learning, which are deficient in this mouse model, were rescued [70,71]. These studies suggest a direct link between Wnt-signaling and autism-like behavior in autism mouse models.

In addition, recent studies in mouse models have determined how autism-related mutations may induce autism-related behaviors through the dysregulation of Wnt-signaling. One such example is *CHD8*, a gene that was recently found to be strongly associated to ASD and encodes for a chromatin-binding protein. One of the first studies on CHD8 in the brain shows that it acts as a positive regulator of the Wnt pathway in the brain [72]. CHD8 binds to the promoter regions of Fzd1, Dvl3 and β-catenin. Knockdown of CHD8 during cortical development results in their downregulation, leading to the reduction of the TCF/LEF transcription factor family and ultimately defective brain development. By expressing a degradation-resistant β-catenin construct in CHD8-downregulated embryos, researchers rescued the aberrant dendritic arborization as well as increased spine density in the CHD8-downregulated mice. In addition, the study showed that inducing stable β-catenin restores the aberrant social and anxiogenic behaviors to the levels seen in control mice. The Shank scaffolding family is an additional group of high-risk ASD genes that have been suggested to interact with Wnt-signaling components. In a recent publication by Harris et al., a Shank-knockout drosophila model was used to study of the potential molecular pathways that the Shank family of genes regulate [73]. Surprisingly, the study revealed that the non-canonical Wnt-Frizzled Nuclear Import (FNI) pathway was affected. In this pathway, Wnt binding induces internalization of the Fz2 receptor, followed by proteolytic cleavage of the receptors’ N-terminus to form Fz2-C, which translocates into the nucleus, where it regulates transcription necessary for synaptic development [74,75]. By knocking out Shank family genes, Harris et al. revealed reduction of the Fz2 internalization into the post-synaptic membrane, reduction of Fz2-C presence in the nucleus, and abnormal synaptic development. Therefore, there is accumulating evidence that autism-associated mutations affect abnormal cortical patterning, synaptic development, and autism-like behaviors through modulation of Wnt-signaling pathway.

## 4. Calcium and Calmodulin Signaling

### 4.1. Roles of Calcium-Signaling and the Calmodulin-Binding Pathway in the Brain

Shifts in Calcium (Ca^2+^) concentrations have been shown to affect the function of several tissues and organs such as the heart, pancreas and components of the Central Nervous System (CNS). Therefore, it is of no surprise that disequilibrium in signaling leads to pathology [76]. In the brain, Ca^2+^ performs specific functions in the presynapse and postsynapse. In the presynapse, arrival of action potentials induce an increase of presynaptic Ca^2+^ levels through activation of the N and P/Q-voltage-gated channel. The influx of Ca^2+^ leads directly to the release of neurotransmitters [77]. However, the role of Ca^2+^ in postsynaptic signal transduction pathways has been found to be dysregulated in the autism genetic studies discussed previously. 

Ca^2+^-induced effects in postsynaptic neuronal function and excitability are mediated through its binding with the protein Calmodulin (CaM; Ca^2+^-Modulated protein), which induces signaling to additional downstream CaM Kinases [77,78]. Glutamic acid induces Ca^2+^ influx at the postsynapse by binding to *N*-methyl-d-aspartate receptors (NMDARs) and metabotropic glutamate receptors such as mGlur1, which initiates the release of internal Ca^2+^ stores [77]. After binding Ca^2+^, CaM may induce the activation of CaMKII or CaMKK2. CaMKII is a dodecamer holoenzyme which has 28 isoforms composed of four different subunits (CaMKIIα through δ), and resides mostly in spines and dendritic shafts of excitatory neurons [79,80,81]. The mandatory role of CaMKII in synaptic function has been well described [82]. Active CaM binds to CaMKII subunits and relieves them from autoinhibition, which leads to autophosphorylation and kinase activity [78]. CaMKIIα kinase activity is mandatory for long-term potentiation (LTP), the main electrophysiological determinant of experience-dependent synaptic strengthening, and is highly involved in behavioral processes, such as learning and memory. Deletion of *CAMKIIA*, or inhibiting the binding of CaM and Ca^2+^ to CaMKII, inhibits LTP induction [83]. CaMKIIβ has been shown to interact with cytoskeleton subunits such as F-actin, α-catinin and the PSD protein Densin-180 for the purpose of spine size regulation, as well as to affect long-term synaptic plasticity by binding and regulating receptors such as GluN2B and GluA1 [80,82]. Furthermore, accumulating evidence show that CaMKII phosphorylates the GABA_A_ β, γ2 and α1 subunits, and regulates GABA_A_ trafficking to the synaptic membrane [84]. Since GABA is the major inhibitory neurotransmitter, these findings highlight that CaMKII plays important roles in both excitatory and inhibitory neurotransmission. 

CaM also activates CaMKK2, whose activity is crucial for spatial memory formation, and the downstream activation of additional kinases: CaMKI is a positive transducer of growth cone motility which is essential for neurite elongation and arborization [85]; phosphorylation of CaMKIV increases gene expression and protein synthesis, and is also necessary for contextual fear [86]. CaMKK2 also phosphorylates other kinases such as the AMP-activated Protein Kinase (AMPK) who’s activity is essential for regulating the energy intake necessary for typical brain function [87]. Interestingly, *CAMKIG* and *CAMKIV* are among the genes which were enriched in the Calmodulin-binding pathway in the Ben-David et al. publication, which looked for common molecular pathways affected by rare and common variations in ASD [40]. Overall, this data provides compelling evidence of how Ca^2+^ signaling and the CaM pathway are involved in neurological functions by affecting a variety of synaptic characteristics, neurotransmission via excitatory and inhibitory receptor regulation and important biological functions such as LTP and LTM. Deficits in the CaM pathway and its branching cascades have the potential to be involved in many neuropsychiatric conditions due to their broad influence on many biological systems, and more specifically by the way it regulates neurotransmission and synaptic characteristics.

### 4.2. How Calcium-Signaling and the Calmodulin-Binding Pathway May Be Involved in ASD

Considering the central role of Ca^2+^ and CaM signaling in synaptic function and neuronal connectivity, it is reasonable to presume that dysregulation of this pathway could lead to autism-related symptoms. However, it is technically challenging to decipher if there are any dysregulation in synaptic functions such as LTP in humans diagnosed with ASD, while evidence for such dysregulation have been frequently observed in several ASD mouse models [88,89]. Therefore, our understanding of the possible role of Ca^2+^ signaling in autism is still at its infancy, compared to more established role of the Wnt pathway. Of great interest, one human study has used Transcranial Magnetic Stimulation (TMS) to study changes in long term potentiation-like synaptic plasticity in humans diagnosed with ASD [90]. In this study, the researchers performed TMS in cortical regions followed by motor-evoked potentials. Individuals with ASD did not show any changes in motor-evoked potentials after TMS, unlike neurotypical controls. This study suggests deficits in plasticity that resemble deficits in LTP. Additional human studies have verified similar deficits in neuronal network connectivity in ASD patients, as has been recently reviewed [91]. These studies have often found changes in electroencephalographic signals after different sensory stimuli in ASD patients. Overall, these studies suggest deficits in synaptic and network activity that may be related to Calcium-signaling. 

While studies of Ca^2+^ signaling in the human brain remains challenging, recent studies have determined disturbances in Ca^2+^ signaling in cells derived from individuals with ASD. Agonist-evoked Ca^2+^ signaling has been shown to be dysfunctional in skin fibroblasts derived from individuals diagnosed with autism [92]. An elegant study was performed on induced Pluripotent Stem Cells (iPSC) derived from individuals diagnosed with Timothy syndrome [93], a syndromic autism where 80% of individuals are diagnosed with ASD. These iPSCs were differentiated into neurons in vitro and displayed dysregulated Ca^2+^ signaling and changes in activity-dependent gene transcription. While these studies suggest that Ca^2+^ signaling and CaM may be involved in the biology of ASD, technological improvements of Ca^2+^ imaging in the human brain, and more high-throughput studies in individuals diagnosed with ASD, are necessary to understand the role of Ca^2+^ in the specific behaviors and brain regions that are particularly relevant to ASD.

Animal models and in vitro studies have given some additional insights into how dysregulation of Ca^2+^ and Calmodulin-binding may be involved in abnormal neurodevelopment. For example, CaMKIV positively regulates the transcription of FMRP (Fragile-X Mental Retardation Protein), the causative gene of FXS [94]. A follow-up study found that a Single Nucleotide Polymorphism (SNP) in the gene CaMKIV (rs25925) is associated with higher risk for ASD development in a European cohort. This SNP appears to be located on a splicing factor binding site, and is predicted to alter the balance of CaMKIV isoforms [95]. In addition, CaMKIIα has been shown to regulate the activity of mGluR5, which is a potential target in FXS treatment [96]. It is still unclear how CaMKIIα perturb mGluR5 activity in FXS, however one proposed mechanism suggests that CaMKIIα is significantly elevated in the synapse of FXS mouse models, which might cause the hyperphosphorylation of the Homer 1 (H1) and 2 (H2) scaffolding proteins, resulting in their dissociation from mGluR5. This dissociation allows the short Homer 1 isoform, H1α, to bind mGluR5 and induce ligand-independent activity of the receptor [97]. Inhibition of mGluR5 in *FMR1* knockout mice improved learning and memory, which highlighted this therapy as a promising pharmaceutical treatment [98]. However, clinical trials that were designed to inhibit mGluR5 and its downstream pathways in FXS patients described only partial success, as extensively reviewed by Schaefer, Davenport and Erickson [99]. In one clinical study, the mGluR5 selective antagonist fenobam induced improvement in prepulse inhibition, but had no effects on the excessive impulsivity observed in FXS patients [100]. In another clinical study, treatment of a small cohort of FXS patients with Lithium—which mitigates signaling pathways activated via mGluR5 signaling—resulted in significant behavioral improvements such in hyperactivity and inappropriate speech, however induced only a tendency for improvement in irritability, lethargy and repetitive behaviors [101]. 

An additional syndromic autism linked with Ca^2+^ signaling dysregulation is Angelman Syndrome (AS), which is characterized by the deletion of the maternal allele of 15q13-11, including the gene *UBE3A*. In the *UBE3A* maternal allele null mouse model, disruptions in the autophosphorylation of CaMKIIα, which is essential for its kinase activity, is responsible for LTP deficits of the hippocampus [102]. CaMKIIα has also been found to interact directly with scaffolding proteins associated with ASD development. Specifically, using immunoprecipitation to pull-down CaMKII from different neuronal cell fractions of the forebrain, it was determined that CaMKII binds Shank3, Dlgap2 and Syngap1 in the synapse [103]. In summary, various studies have identified CaMKIV and CaMKIIα as the two main Ca^2+^ signaling enzymes that are most likely to be involved in ASD. On one hand the Ca^2+^ and Calmodulin-binding pathways may be interesting targets for therapeutic interventions. However, future studies in mouse models, and electrophysiology studies in humans, are necessary to understand how and why the Ca^2+^ signaling pathway is involved in ASD. Further studies are particularly needed to clarify the potential roles of Ca^2+^ signaling in social behaviors or repetitive behaviors, which form the core features of ASD. 

## 5. Additional Signaling Pathways

Thus far, we have discussed the Wnt-signaling and Calmodulin-binding pathways as potential molecular mechanisms that are involved in ASD, due to the fact that these pathways have been found to be enriched for ASD-associated genes in multiple network-based analysis. In addition to these pathways, the mTOR-signaling and NGF-signaling pathways have also appeared in some of the discussed bioinformatic publications, although not quite as often as the Wnt and Calmodulin pathways. The mTOR pathway has already gained significant attention in connection to autism due to its significant involvement in syndromic autisms, as explained below. There is yet little known connection between NGF and ASD, however it is worth shortly considering the possible connections, considering its roles in brain development and function.

### 5.1. The PI3K/Mtor-Signaling Pathway

Mammalian Target of Rapamycin (mTOR) is a serine/threonine kinase, which is considered a central kinase in organism development. Therefore it is of no surprise that ablation of mTOR results in in-utero death a short time after the implantation stage of the embryo in the uterus endometrium [104]. The mTOR pathway regulates brain development through two main cascades driven by two complexes: mTOR complex 1 (mTORC1) and mTOR complex 2 (mTORC2). In the developing brain each cascade seems to take part in unique developmental duties [105]. The mTORC2 cascade facilitates growth cone motility through its interaction with actin filaments. This role is essential for pathfinding dynamics of the neurite in the developing brain. Additionally, mTORC2 has an indirect influence on neuron size and morphology by regulating mTORC1 through RAC-alpha serine/threonine-protein kinase (AKT) activity. AKT activates mTORC1 which in turn phosphorylates p70 ribosomal protein S6 kinase (p70S6K) and eukaryotic Initiation Factor 4E (eIF4E)-binding protein (4EBP), which enhance a downstream cascade necessary for both protein synthesis and lipid synthesis. The products are used for plasma membrane expansion that is required for neurite elongation and arborization, and dendrite formation. This developmental mechanism driven by mTORC1 may also be directly activated by extracellular stimulation such as growth factors and neurotransmitters.

There is an abundance of publications linking mTOR-signaling to ASD, and it is considered to be one of the most promising converging signaling pathway candidates for ASD development. The involvement of mTOR-signaling in ASD and other neuropsychiatric conditions has been thoroughly reviewed previously [106]. However, unlike the previously described pathways in this review, and to the best of our knowledge, only one publication has described a link between the mTOR-signaling pathway and ASD using a network analysis-based approach [38]. Rather, the evidence for mTOR-signaling as a promising common ASD pathway candidate arise mostly from in vivo research into syndromic autisms such as Tuberous Sclerosis (TS), *PTEN*-related syndrome, Neurofibromatosis Type 1 (NF-1) and FXS [12,107,108,109]. In TS, the TSC1/2 complex has an inhibitory role on mTORC1, and knockdown of TSC2 results in over-activation of mTORC1 and mTORC2, leading to an increase in neuronal cell size of the fetal brain [110]. Interestingly, blocking mTOR activity in TSC2-haploinsufficient mice using rapamycin reversed social deficits, suggesting that mTOR-signaling has a role in the manifestation of overall autistic behaviors, and in TSC particularly [111]. PTEN is a phosphatase which also acts as a negative regulator of the mTOR-signaling pathway by reducing the activity of the PI3K/AKT pathway. As previously mentioned, AKT activates mTORC1 which is essential for protein synthesis of the developing neurite. Therefore, PTEN dysfunction might lead to increased protein synthesis and abnormal brain growth. Indeed, it has been shown that *PTEN* mutations in a subset of ASD cases are co-morbid with overgrowth and macrocephaly [112]. Mouse models with conditional knockout of *PTEN* in mature neurons of the cerebral cortex and hippocampus display axonal overgrowth, ectopic axonal projections, and abnormal synapses, as well as reduced social interaction, increased anxiety, and hyperexcitability [113]. In a recent publication by Cupolillo et al., conditional knockout of PTEN in cerebellar Purkinje cells led to a reduction in social interaction and repetitive behavior, paralleled with structural abnormalities in cerebellar axons and dendrites [114]. Neurofibromin (NF), a tumor suppressor through its GTPase-activating function, is an additional negative regulator of mTOR-signaling via TSC2 [115]. It is the key inducer of the familial cancer syndrome, NF-1, a pathology where autism-like social dysfunction has also been observed [108]. It is still unclear exactly how NF is specifically involved with the ASD-like phenotype observed in NF-1. However, considering that the disequilibrium of the negative regulation of mTOR-signaling both by TSC1/2 and PTEN has been shown to be impaired in syndromic autisms, it is possible to claim that NF has a role in ASD-like phonotype development through its regulation of mTOR. In the case of FXS, increased phosphorylation of mTOR and p70S6K was observed both in lymphocytes and brain samples of FXS patients, which suggest increased protein synthesis, which is the main avenue by which mTOR dysregulation is believe to induce ASD development [109].

In all of the examples given above, inability to downregulate mTOR-signaling is associated with the autism phenotype. In fact, studies determined that blocking mTOR through the use of Rapamycin improves social deficits in the TS and BTBR mouse models [111,116] which implies that Rapamycin might be a possible therapy for the social deficits in ASD.

### 5.2. Nerve Growth Factor

Nerve Growth Factor (NGF) is a primary neurotrophin for peripheral organ innervation and sensory neuron development. As the nervous system develops, target-organs secret NGF that is detected by elongating axons, and through receptor-mediated endocytosis it enhances survival, neurite outgrowth and synaptic plasticity and connectivity [117]. A role for NGF in the CNS was initially observed in the developing rat forebrain, as researchers injected exogenous NGF intraventricularly to neonatal rats, which resulted in an increase of Choline Acetyltransferase (ChAT) [118]. Further studies of NGF in the rat forebrain showed that NGF regulates cholinergic development and differentiation by binding Tropomyosin receptor kinase A (TrkA), which by a positive feedback loop, increases TrkA expression as well as ChAT in cholinergic neurons [119]. Since then, NGF, together with BDNF, have been shown to play an important role in orchestrating neuronal plasticity important for sociability in mice [117]. 

Given its important role in nervous system development, it is not surprising that dysregulation in NGF-signaling has been implicated in psychiatric disorders such as depression, schizophrenia and Alzheimer’s disease [117]. Nevertheless, the number of publications linking NGF to ASD is scarce. In fact, some publications have shown that NGF levels in cerebral spinal fluid (CSF) and blood is typical in children with ASD [120,121]. However, more recent publications begin to present a different picture. A study analyzing Differential Alternative Splicing (DAS) in the blood mRNA of 2–4 year old boys diagnosed with ASD showed there was a significant difference in DAS for several genes involved in NGF-signaling, including the NGF receptors, Nerve Growth Factor Receptor (NGFR) and Neurotrophic Receptor Tyrosine Kinase 1 (NTRK1) [122]. A different study showed that SNPs in NTRK1 associated with ASD behavioral traits measured by the Empathy Quotient (EQ) and the Autism Spectrum Quotient (AQ) [123]. In an interesting study by Lu et al., researchers conducted a genome-wide Quantitative Trait Loci (QTL) study and found that several SNPs in NGF are significantly associated with deficits in non-verbal communication, which is an autistic trait [124]. The reasoning for the QTL approach was to focus on a specific trait and find the genetic loci associated to it and reduce some of the genetic heterogeneity that usually complicates ASD genetic research. Therefore, while there is scarce evidence for the role of NGF-signaling in ASD from animal studies, human genetic studies have actually found associations between the NGF pathway and behaviors that are dysregulated in ASD. Therefore, further research into the possible mechanistic roles for NGF in social behavior are needed [39].

## 6. Conclusions

ASD’s elusive genetic etiology imposes a great challenge to our understanding of the disorder’s pathology. The growing number of genes associated with ASD, both rare and common variants, and the fact that these genes are involved in a variety of biological processes, makes it difficult for the research community to find a specific target for therapy. Taking into account that there are currently no pharmacological agents that treat the core symptoms of ASD, there is great need to understand the biological pathways that are targeted by ASD-mutations, which can be novel pharmacological targets. Both Wnt and the Ca^2+^ signaling pathways that we discussed in-depth in this review are potential targets for pharmacological intervention. However, while these pathways have been well characterized, there is still a great need to understand exactly how dysregulation of these pathways are involved in the core characteristics of ASD, including social and repetitive behaviors. A more clear understanding of the specific roles of these pathways in specific brain region is also likely to shed some light into this issue.

Up until now, the only way to find common pathways affected by ASD-associated genes was by in-silico network analysis. However, with the creation of multiple mouse models based on these genetic aberrations, a current method can be to decipher common molecular dysregulations found in these multiple autism mouse models, and to correlate these dysregulations with the animal’s behavioral and neurodevelopmental phenotypes. For example, Ellegood et al. revealed that autism mouse models can be clustered according to neuroanatomical differences such as changes in the volume of different brain regions [125]. In a recent review, Kim et al., compared publications on multiple mouse models, and searched for physiological dysregulations underlying the variety of repetitive behavior types observed in ASD mouse models. While Kim et al. deciphered that it is difficult to link repetitive behavior types to specific brain regions, they discovered a few common pathways that are often involved in ASD phenotypes [126]. One example is involvement of glutamatergic connections from the frontal cortex to the midbrain, involved in repetitive behaviors. Therefore, the spatial and temporal resolution of ASD-related molecular pathways cannot be determined by network analysis of genetic data, but rather through the analysis of in vivo models. Therefore, parallel investigation of molecular mechanisms dysregulated in multiple mouse models of autism genes is likely to reveal important mechanisms and novel therapeutic targets.

## Abbreviations

ADHD	Attention Deficit Hyperactivity Disorder
AKT	RAC-alpha serine/threonine-protein kinase
AMPK	AMP-activated Protein Kinase
AQ	Autism Spectrum Quotient
AS	Angelman Syndrome
ASD	Autism Spectrum Disorder
BDNF	Brain-derived Neurotrophic Factor
BIO	(2′*Z*,3′*E*)-6-Bromoindirubin-3′-oxime
CaM	Calmodulin
CAMK	Ca^2+^/calmodulin-dependent protein kinase
CASK	Calcium/Calmodulin Dependent Serine Protein Kinase
CDFE	Cortical Dysplasia-focal Epilepsy
ChAT	Choline Acetyltransferase
CHD8	Chromodomain Helicase DNA Binding Protein 8
CNS	Central Nervous System
CNTNAP2	Contacting Associated Protein 2
CNV	Copy Number Variation
COL11A1	Collagen Type XI Alpha 1 Chain
DAS	Differential Alternative Splicing
Dlgap2	DLG Associated Protein 2
Dvl3	Dishevelled Segment Polarity Protein 3
eIF4E	Eukaryotic Translation Initiation Factor 4E
EQ	Empathy Quotient
FMR1	Fragile-X Mental Retardation 1
FNI	Frizzled Nuclear Import
FOXP1	Forkhead Box P1
FXS	Fragile-X Syndrome
Fzd1	Frizzled Class Receptor 1
GABA	Gamma-Aminobutyric
GJB6	Gap Junction Protein Beta 6
GOC	Gene Ontology Consortium
GRIN2B	Glutamate Ionotropic Receptor NMDA Type Subunit 2B
GSK3β	Glycogen Synthase Kinase 3 Beta
GWAS	Genome Wide Association Study
H1	Homer 1
H2	Homer 2
Hipk1	Homeodomain Interacting Protein Kinase 1
ID	Intellectual Deficiency
IPA	Ingenuity Pathway Analysis
iPSC	induced pluripotent stem cells
KATNAL2	Katanin Catalytic Subunit A1 Like 2
KCNQ4	Potassium Voltage-Gated Channel Subfamily Q Member 4
KEGG	Kyoto Encyclopedia of Genes and Genomes
LAMC3	Laminin Subunit Gamma 3
LEF	Lymphoid Enhancer Binding Factor
LRP5	LDL Receptor Related Protein 5
LRP6	LDL Receptor Related Protein 6
LTP	Long Term Potentiation
MECP2	Methyl CpG binding protein 2
mGluR5	Glutamate Metabotropic Receptor 5
mTOR	Mammalian Target of Rapamycin
mTORC1	Mammalian Target of Rapamycin complex 1
mTORC2	Mammalian Target of Rapamycin complex 2
MYH14	Myosin Heavy Chain 14
NF	Neurofibromin
NF-1	Neurofibromatosis Type 1
NGF	Nerve Growth Factor
NGFR	Nerve Growth Factor Receptor
NGS	Next Generations Sequencing
NMDAR	*N*-methyl-d-aspartate receptors
NSC	Neuronal Stem Cell
NTNG1	Netrin G1
NTRK1	Neurotrophic Receptor Tyrosine Kinase 1
p70S6K	p70 ribosomal protein S6 kinase
PI3K	Phosphatidylinositol-4,5-Bisphosphate 3-Kinase
PPI	Protein-Protein Interaction
PROSAP2	Proline Rich Synapse Associated Protein 2
PSD	Post Synaptic Density
PTEN	Phosphatase And Tensin Homolog
PV	Parvalbumin
PVD	Pial Vessel Disruption
QTL	Quantitative Trait Loci
SCN1A	Sodium Voltage-Gated Channel Alpha Subunit 1
SCN2A	Sodium Voltage-Gated Channel Alpha Subunit 2
SFARI	Simmons Foundation Autism Research Initiative
SHANK3	SH3 And Multiple Ankyrin Repeat Domains 3
SNP	Single Nucleotide Polymorphism
STXBP1	Syntaxin Binding Protein 1
SVZ	Subventricular Zone
Syngap1	Synaptic Ras GTPase Activating Protein 1
TCF	Transcription Factor
THRA	Thyroid Hormone Receptor, Alpha
TMS	Transcranial Magnetic Stimulation
TrkA	Tropomyosin receptor kinase A
TSC1	Tuberous Sclerosis 1
TSC2	Tuberous Sclerosis 2
UBE3A	Ubiquitin Protein Ligase E3A
WGCNA	Weighted Gene Co-expression Network Analysis
Wnt	Wingless-type

## References

[1] Grice D.E., Buxbaum J.D. (2006). The Genetics of Autism Spectrum Disorders. NeuroMol. Med..

[2] Folstein S., Rutter M. (1977). Infantile autism: A genetic study of 21 twin pairs. J. Child Psychol. Psychiatry.

[3] Folstein S., Rutter M., Rutter M., Schopler E. (1978). A Twin Study of Individuals with Infantile Autism. Autism: A Reappraisal of Concepts and Treatment.

[4] Rutter M. (2016). Heritability of autism spectrum disorders: A meta-analysis of twin studies. J. Child Psychol. Psychiatry.

[5] Taurines R., Schwenck C., Westerwald E., Sachse M., Siniatchkin M., Freitag C. (2012). ADHD and autism: Differential diagnosis or overlapping traits? A selective review. Atten. Defict Hyperact. Disord..

[6] Hollocks M.J., Howlin P., Papadopoulos A.S., Khondoker M., Simonoff E. (2014). Differences in HPA-axis and heart rate responsiveness to psychosocial stress in children with autism spectrum disorders with and without co-morbid anxiety. Psychoneuroendocrinology.

[7] Talkowski M.E., Mullegama S.V., Rosenfeld J.A., Van Bon B.W.M., Shen Y., Repnikova E.A., Gastier-Foster J., Thrush D.L., Kathiresan S., Ruderfer D.M. (2011). Assessment of 2q23.1 microdeletion syndrome implicates MBD5 as a single causal locus of intellectual disability, epilepsy, and autism spectrum disorder. Am. J. Hum. Genet..

[8] Khanzada N., Butler M., Manzardo A. (2017). GeneAnalytics Pathway Analysis and Genetic Overlap among Autism Spectrum Disorder, Bipolar Disorder and Schizophrenia. Int. J. Mol. Sci..

[9] Buxbaum J.D., Hof P.R. (2012). The Neuroscience of Autism Spectrum Disorders.

[10] Hagerman R., Hoem G., Hagerman P. (2010). Fragile X and autism: Intertwined at the molecular level leading to targeted treatments. Mol. Autism.

[11] Strauss K.A., Puffenberger E.G., Huentelman M.J., Gottlieb S., Dobrin S.E., Parod J.M., Stephan D.A., Morton D.H. (2006). Recessive symptomatic focal epilepsy and mutant contactin-associated protein-like 2. N. Engl. J. Med..

[12] Vignoli A., La Briola F., Peron A., Turner K., Vannicola C., Saccani M., Magnaghi E., Scornavacca G.F., Canevini M.P. (2015). Autism spectrum disorder in tuberous sclerosis complex: Searching for risk markers. Orphanet J. Rare Dis..

[13] Percy A.K. (2011). Rett syndrome: Exploring the autism link. Arch. Neurol..

[14] Leppa V.M., Kravitz S.N., Martin C.L., Andrieux J., Le Caignec C., Martin-Coignard D., DyBuncio C., Sanders S.J., Lowe J.K., Cantor R.M. (2016). Rare Inherited and De Novo CNVs Reveal Complex Contributions to ASD Risk in Multiplex Families. Am. J. Hum. Genet..

[15] Wilson H.L. (2003). Molecular characterisation of the 22q13 deletion syndrome supports the role of haploinsufficiency of SHANK3/PROSAP2 in the major neurological symptoms. J. Med. Genet..

[16] Durand C.M., Betancur C., Boeckers T.M., Bockmann J., Chaste P., Fauchereau F., Nygren G., Rastam M., Gillberg I.C., Anckarsäter H. (2007). Mutations in the gene encoding the synaptic scaffolding protein SHANK3 are associated with autism spectrum disorders. Nat. Genet..

[17] Wang X., Xu Q., Bey A.L., Lee Y., Jiang Y.-H. (2014). Transcriptional and functional complexity of SHANK3 provides a molecular framework to understand the phenotypic heterogeneity of SHANK3 causing autism and SHANK3 mutant mice. Mol. Autism.

[18] Ornoy A., Liza W.F., Ergaz Z. (2016). Genetic syndromes, maternal diseases and antenatal factors associated with autism spectrum disorders (ASD). Front. Neurosci..

[19] De Anda F.C., Rosario A.L., Durak O., Tran T., Gräff J., Meletis K., Rei D., Soda T., Madabhushi R., Ginty D.D. (2012). Autism spectrum disorder susceptibility gene TAOK2 affects basal dendrite formation in the neocortex. Nat. Neurosci..

[20] Golzio C., Willer J., Talkowski M.E., Oh E.C., Taniguchi Y., Jacquemont S., Reymond A., Sun M., Sawa A., Gusella J.F. (2012). KCTD13 is a major driver of mirrored neuroanatomical phenotypes of the 16p11.2 copy number variant. Nature.

[21] O’Roak B.J., Deriziotis P., Lee C., Vives L., Schwartz J.J., Girirajan S., Karakoc E., Mackenzie A.P., Ng S.B., Baker C. (2011). Exome sequencing in spordic autism spectrum disorders identifies severe de novo mutations. Nat. Genet..

[22] Iossifov I., Ronemus M., Levy D., Wang Z., Hakker I., Rosenbaum J., Yamrom B., Lee Y.H., Narzisi G., Leotta A. (2012). De Novo Gene Disruptions in Children on the Autistic Spectrum. Neuron.

[23] O’Roak B.J., Vives L., Girirajan S., Karakoc E., Krumm N., Coe B.P., Levy R., Ko A., Lee C., Smith J.D. (2012). Sporadic autism exomes reveal a highly interconnected protein network of de novo mutations. Nature.

[24] Sanders S.J., Murtha M.T., Gupta A.R., Murdoch J.D., Raubeson M.J., Willsey A.J., Ercan-Sencicek A.G., DiLullo N.M., Parikshak N.N., Stein J.L. (2012). De novo mutations revealed by whole-exome sequencing are strongly associated with autism. Nature.

[25] Yuen R.K.C., Thiruvahindrapuram B., Merico D., Walker S., Tammimies K., Hoang N., Chrysler C., Nalpathamkalam T., Pellecchia G., Liu Y. (2015). Whole-genome sequencing of quartet families with autism spectrum disorder. Nat. Med..

[26] Gaugler T., Klei L., Sanders S.J., Bodea C.A., Goldberg A.P., Lee A.B., Mahajan M., Manaa D., Pawitan Y., Reichert J. (2014). Most genetic risk for autism resides with common variation. Nat. Genet..

[27] Basu S.N., Kollu R., Banerjee-Basu S. (2009). AutDB: A gene reference resource for autism research. Nucleic Acids Res..

[28] Geschwind D.H. (2008). Autism: Many genes, common pathways?. Cell.

[29] Parikshak N.N., Gandal M.J., Geschwind D.H. (2015). Systems biology and gene networks in neurodevelopmental and neurodegenerative disorders. Nat. Rev. Genet..

[30] Ashburner M., Ball C.A., Blake J.A., Botstein D., Butler H., Michael Cherry J., Davis A.P., Dolinski K., Dwight S.S., Eppig J.T., The Gene Ontology Consortium (2000). Gene Ontology: Tool for the unification of biology. Nat. Genet..

[31] Chowdhury S., Sarkar R.R. (2015). Comparison of human cell signaling pathway databases—Evolution, drawbacks and challenges. Database.

[32] Ogata H., Goto S., Sato K., Fujibuchi W., Bono H., Kanehisa M. (1999). KEGG: Kyoto Encyclopedia of Genes and Genomes. Nucleic Acids Res..

[33] Ingenuity Systems Ingenuity Pathway Analysis (IPA). https://www.qiagenbioinformatics.com/.

[34] Mines M.A., Yuskaitis C.J., King M.K., Beurel E., Jope R.S. (2010). GSK3 Influences Social Preference and Anxiety-Related Behaviors during Social Interaction in a Mouse Model of Fragile X Syndrome and Autism. PLoS ONE.

[35] Okerlund N.D., Cheyette B.N.R. (2011). Synaptic Wnt signaling—A contributor to major psychiatric disorders?. J. Neurodev. Disord..

[36] Iossifov I., O’roak B.J., Sanders S.J., Ronemus M., Krumm N., Levy D., Stessman H.A., Witherspoon K., Vives L., Patterson K.E. (2014). The contribution of de novo coding mutations to autism spectrum disorder. November.

[37] Hadley D., Wu Z.-L., Kao C., Kini A., Mohamed-Hadley A., Thomas K., Vazquez L., Qiu H., Mentch F., Pellegrino R. (2014). The impact of the metabotropic glutamate receptor and other gene family interaction networks on autism. Nat. Commun..

[38] Wen Y., Alshikho M.J., Herbert M.R. (2016). Pathway network analyses for autism reveal multisystem involvement, major overlaps with other diseases and convergence upon MAPK and Calcium signaling. PLoS ONE.

[39] Lin G.N., Corominas R., Lemmens I., Yang X., Tavernier J., Hill D.E., Vidal M., Sebat J., Iakoucheva L.M. (2015). Spatiotemporal 16p11.2 Protein Network Implicates Cortical Late Mid-Fetal Brain Development and KCTD13-Cul3-RhoA Pathway in Psychiatric Diseases. Neuron.

[40] Ben-David E., Shifman S. (2012). Networks of neuronal genes affected by common and rare variants in autism spectrum disorders. PLoS Genet..

[41] MacDonald B.T., Tamai K., He X. (2009). Wnt/β-Catenin Signaling: Components, Mechanisms, and Diseases. Dev. Cell.

[42] Polakis P. (2000). Wnt signaling and cancer. Genes Dev..

[43] Rao T.P., Kühl M. (2010). An updated overview on Wnt signaling pathways: A prelude for more. Circ. Res..

[44] Noelanders R., Vleminckx K. (2016). How Wnt Signaling Builds the Brain. Neuroscientist.

[45] Chen A., Walsh C. (2002). Regulation of Cerebral Cortical Size by Control of Cell Cycle Exit in Neural Precursors. Science.

[46] Woodhead G.J., Mutch C.A., Olson E.C., Chenn A. (2006). Cell-Autonomous beta-Catenin Signaling Regulates Cortical Precursor Proliferation. J. Neurosci..

[47] Duncan R.N., Xie Y., McPherson A.D., Taibi A.V., Bonkowsky J.L., Douglass A.D., Dorsky R.I. (2015). Hypothalamic radial glia function as self-renewing neural progenitors in the absence of Wnt/β-catenin signaling. Development.

[48] Piccin D., Morshead C.M. (2011). Wnt signaling regulates symmetry of division of neural stem cells in the adult brain and in response to injury. Stem Cells.

[49] Marinaro C., Pannese M., Weinandy F., Sessa A., Bergamaschi A., Taketo M.M., Broccoli V., Comi G., Götz M., Martino G. (2012). Wnt signaling has opposing roles in the developing and the adult brain that are modulated by Hipk1. Cereb. Cortex.

[50] Huber O., Krohn M., Kemler R. (1997). A specific domain in alpha-catenin mediates binding to beta-catenin or plakoglobin. J. Cell Sci..

[51] Huber A.H., Weis W.I. (2001). The structure of the B-catenin/E-cadherin complex and the molecular basis of diverse ligand recognition by β-catenin. Cell.

[52] Yu X., Malenka R.C. (2003). Beta-catenin is critical for dendritic morphogenesis. Nat. Neurosci..

[53] Bian W.-J., Miao W.-Y., He S.-J., Qiu Z., Yu X. (2015). Coordinated Spine Pruning and Maturation Mediated by Inter-Spine Competition for Cadherin/Catenin Complexes. Cell.

[54] Murase S., Mosser E., Schuman E.M. (2002). Depolarization drives β-catenin into neuronal spines promoting changes in synaptic structure and function. Neuron.

[55] Adams C.L., Nelson W.J., Smith S.J. (1996). Quantitative Analysis of Cadherin-Catenin-Actin Reorganization during Development of Cell-Cell Adhesion. J. Cell Biol..

[56] Bamji S.X., Shimazu K., Kimes N., Huelsken J., Birchmeier W., Lu B., Reichardt L.F. (2003). Role of β-catenin in synaptic vesicle localization and presynaptic assembly. Neuron.

[57] Butz S., Okamoto M., Südhof T.C. (1998). A Tripartite Protein Complex with the Potential to Couple Synaptic Vesicle Exocytosis to Cell Adhesion in Brain. Cell.

[58] Han G.A., Malintan N.T., Collins B.M., Meunier F.A., Sugita S. (2010). Munc18-1 as a key regulator of neurosecretion. J. Neurochem..

[59] Bolshakov V.Y., Siegelbaum S.A. (1995). Regulation of hippocampal transmitter release during development and long-term potentiation. Science.

[60] Taylor A.M., Wu J., Tai H.C., Schuman E.M. (2013). Axonal translation of beta-catenin regulates synaptic vesicle dynamics. J. Neurosci..

[61] Sharma K., Choi S.Y., Zhang Y., Nieland T.J.F., Long S., Li M., Huganir R.L. (2013). High-throughput genetic screen for synaptogenic factors: Identification of LRP6 as critical for excitatory synapse development. Cell Rep..

[62] Hutsler J.J., Zhang H. (2010). Increased dendritic spine densities on cortical projection neurons in autism spectrum disorders. Brain Res..

[63] Stoner R., Chow M.L., Boyle M.P., Sunkin S.M., Mouton P.R., Roy S., Wynshaw-Boris A., Colamarino S.A., Lein E.S., Courchesne E. (2014). Patches of disorganization in the neocortex of children with autism. N. Engl. J. Med..

[64] Casanova M.F., van Kooten I., Switala A.E., van Engeland H., Heinsen H., Steinbusch H.W.M., Hof P.R., Schmitz C. (2006). Abnormalities of cortical minicolumnar organization in the prefrontal lobes of autistic patients. Clin. Neurosci. Res..

[65] Chung K., Wallace J., Kim S.-Y., Kalyanasundaram S., Andalman A.S., Davidson T.J., Mirzabekov J.J., Zalocusky K.A., Mattis J., Denisin A.K. (2013). Structural and molecular interrogation of intact biological systems. Nature.

[66] Dong F., Jiang J., McSweeney C., Zou D., Liu L., Mao Y. (2016). Deletion of CTNNB1 in inhibitory circuitry contributes to autism-associated behavioral defects. Hum. Mol. Genet..

[67] Mines M.A., Jope R.S. (2011). Glycogen synthase kinase-3: A promising therapeutic target for fragile X syndrome. Front. Mol. Neurosci..

[68] Caracci M.O., Avila M.E., de Ferrari G.V. (2016). Synaptic Wnt/GSK3β-Signaling Hub in Autism. Neural Plast..

[69] Min W.W., Yuskaitis C.J., Yan Q., Sikorski C., Chen S., Jope R.S., Bauchwitz R.P. (2009). Elevated glycogen synthase kinase-3 activity in Fragile X mice: Key metabolic regulator with evidence for treatment potential. Neuropharmacology.

[70] Guo W., Murthy A.C., Zhang L., Johnson E.B., Schaller E.G., Allan A.M., Zhao X. (2012). Inhibition of GSK3β improves hippocampusdependent learning and rescues neurogenesis in a mouse model of fragile X syndrome. Hum. Mol. Genet..

[71] Franklin A.V., King M.K., Palomo V., Martinez A., Mcmahon L.L., Jope R.S. (2014). Glycogen synthase kinase-3 inhibitors reverse deficits in long- term potentiation and cognition in Fragile X mice. Biol. Psychiatry.

[72] Durak O., Gao F., Kaeser-Woo Y., Rueda R., Martorell A., Nott A., Liu C., Watson L., Tsai L.-H. (2016). Chd8 mediates cortical neurogenesis via transcriptional regulation of cell cycle and Wnt signaling. Nat. Neurosci..

[73] Harris K.P., Akbergenova Y., Cho R.W., Baas-Thomas M.S., Littleton J.T. (2016). Shank Modulates Postsynaptic Wnt Signaling to Regulate Synaptic Development. J. Neurosci..

[74] Budnik V., Salinas P.C. (2011). Wnt signaling during synaptic development and plasticity. Curr. Opin. Neurobiol..

[75] Mathew D., Ataman B., Chen J., Zhang Y., Cumberledge S., Budnik V. (2005). Wingless Signaling at Synapses Is through Cleavage and Nuclear Import of Receptor DFrizzled2. Science.

[76] Carafoli E. (2004). Special issue: Calcium signaling and disease. Biochem. Biophys. Res. Commun..

[77] Berridge M.J., Lipp P., Bootman M.D. (2000). The versatility and universality of Calcium signalling. Nat. Rev. Mol. Cell Biol..

[78] Clapham D.E. (2007). Calcium Signaling. Cell.

[79] Bossuyt J., Bers D.M. (2013). Visualizing CaMKII and CaM activity: A paradigm of compartmentalized signaling. J. Mol. Med..

[80] Okamoto K.-I., Narayanan R., Lee S.H., Murata K., Hayashi Y. (2007). The role of CaMKII as an F-actin-bundling protein crucial for maintenance of dendritic spine structure. Proc. Natl. Acad. Sci. USA.

[81] Jalan-Sakrikar N., Bartlett R.K., Baucum A.J., Colbran R.J. (2012). Substrate-selective and calcium-independent activation of CaMKII by α-actinin. J. Biol. Chem..

[82] Hell J.W. (2014). CaMKII: Claiming center stage in postsynaptic function and organization. Neuron.

[83] Lisman J., Schulman H., Cline H. (2002). The molecular basis of CaMKII function in synaptic and behavioural memory. Nat. Rev. Neurosci..

[84] Houston C.M., He Q., Smart T.G. (2009). CaMKII phosphorylation of the GABA A receptor: Receptor subtype-and synapse-specific modulation. J. Physiol..

[85] Wayman G.A., Kaech S., Grant W.F., Davare M., Impey S., Tokumitsu H., Nozaki N., Banker G., Soderling T.R. (2004). Regulation of axonal extension and growth cone motility by calmodulin-dependent protein kinase I. J. Neurosci..

[86] Mizuno K., Ris L., Sánchez-Capelo A., Godaux E., Giese K.P. (2006). Ca^2+^/calmodulin kinase kinase alpha is dispensable for brain development but is required for distinct memories in male, though not in female, mice. Mol. Cell. Biol..

[87] Marcelo K.L., Means A.R., York B. (2016). The Ca^2+^/Calmodulin/CaMKK2 Axis: Nature’s Metabolic CaMshaft. Trends Endocrinol. Metab..

[88] Yun S.H., Trommer B.L. (2011). Fragile X mice: Reduced long-term potentiation and *N*-Methyl-d-Aspartate receptor-mediated neurotransmission in dentate gyrus. J. Neurosci. Res..

[89] Moretti P., Levenson J.M., Battaglia F., Atkinson R., Teague R., Antalffy B., Armstrong D., Arancio O., Sweatt J.D., Zoghbi H.Y. (2006). Learning and memory and synaptic plasticity are impaired in a mouse model of Rett syndrome. J. Neurosci..

[90] Jung N.H., Janzarik W.G., Delvendahl I., Münchau A., Biscaldi M., Mainberger F., Bäumer T., Rauh R., Mall V. (2013). Impaired induction of long-term potentiation-like plasticity in patients with high-functioning autism and Asperger syndrome. Dev. Med. Child Neurol..

[91] Modi M.E., Sahin M. (2017). Translational use of event-related potentials to assess circuit integrity in ASD. Nat. Rev. Neurol..

[92] Schmunk G., Nguyen R.L., Ferguson D.L., Kumar K., Parker I., Gargus J.J. (2017). High-throughput screen detects calcium signaling dysfunction in typical sporadic autism spectrum disorder. Sci. Rep..

[93] Paşca S.P., Portmann T., Voineagu I., Yazawa M., Shcheglovitov A., Paşca A.M., Cord B., Palmer T.D., Chikahisa S., Nishino S. (2011). Using iPSC-derived neurons to uncover cellular phenotypes associated with Timothy syndrome. Nat. Med..

[94] Wang H., Wu L.-J.J., Zhang F., Zhuo M. (2008). Roles of calcium-stimulated adenylyl cyclase and calmodulin-dependent protein kinase {IV} in the regulation of {FMRP} by group I metabotropic glutamate receptors. J. Neurosci..

[95] Waltes R., Duketis E., Knapp M., Anney R.J.L., Huguet G., Schlitt S., Jarczok T.A., Sachse M., Kämpfer L.M., Kleinböck T. (2014). Common variants in genes of the postsynaptic FMRP signalling pathway are risk factors for autism spectrum disorders. Hum. Genet..

[96] Pop A.S., Gomez-Mancilla B., Neri G., Willemsen R., Gasparini F. (2014). Fragile X syndrome: A preclinical review on metabotropic glutamate receptor 5 (mGluR5) antagonists and drug development. Psychopharmacology.

[97] Guo W., Ceolin L., Collins K.A., Perroy J., Huber Correspondence K.M., Huber K.M. (2015). Elevated CaMKIIα and Hyperphosphorylation of Homer Mediate Circuit Dysfunction in a Fragile X Syndrome Mouse Model. Cell Rep..

[98] Michalon A., Bruns A., Risterucci C., Honer M., Ballard T.M., Ozmen L., Jaeschke G., Wettstein J.G., von Kienlin M., Künnecke B. (2013). Chronic Metabotropic Glutamate Receptor 5 Inhibition Corrects Local Alterations of Brain Activity and Improves Cognitive Performance in Fragile X Mice. Biol. Psychiatry.

[99] Schaefer T.L., Davenport M.H., Erickson C.A. (2015). Emerging pharmacologic treatment options for fragile X syndrome. Appl. Clin. Genet..

[100] Berry-Kravis E., Hessl D., Coffey S., Hervey C., Schneider A., Yuhas J., Hutchison J., Snape M., Tranfaglia M., Nguyen D.V. (2009). A pilot open label, single dose trial of fenobam in adults with fragile X syndrome. J. Med. Genet..

[101] Berry-Kravis E., Sumis A., Hervey C., Nelson M., Porges S.W., Weng N., Weiler I.J., Greenough W.T. (2008). Open-label treatment trial of lithium to target the underlying defect in fragile X syndrome. J. Dev. Behav. Pediatr..

[102] Weeber E.J., Jiang Y.-H., Elgersma Y., Varga A.W., Carrasquillo Y., Brown S.E., Christian J.M., Mirnikjoo B., Silva A., Beaudet A.L. (2003). Derangements of hippocampal calcium/calmodulin-dependent protein kinase II in a mouse model for Angelman mental retardation syndrome. J. Neurosci..

[103] Baucum A.J., Shonesy B.C., Rose K.L., Colbran R.J. (2015). Quantitative Proteomics Analysis of CaMKII Phosphorylation and the CaMKII Interactome in the Mouse Forebrain. ACS Chem. Neurosci..

[104] Murakami M., Ichisaka T., Maeda M., Oshiro N., Hara K., Edenhofer F., Kiyama H., Yonezawa K., Yamanaka S. (2004). mTOR is essential for growth and proliferation in early mouse embryos and embryonic stem cells. Mol. Cell. Biol..

[105] Takei N., Nawa H. (2014). mTOR signaling and its roles in normal and abnormal brain development. Front. Mol. Neurosci..

[106] Chen J.A., Peñagarikano O., Belgard T.G., Swarup V., Geschwind D.H. (2015). The Emerging Picture of Autism Spectrum Disorder: Genetics and Pathology. Annu. Rev. Pathol. Mech. Dis..

[107] McBride K.L., Varga E.A., Pastore M.T., Prior T.W., Manickam K., Atkin J.F., Herman G.E. (2010). Confirmation study of PTEN mutations among individuals with autism or developmental delays/mental retardation and macrocephaly. Autism Res..

[108] Plasschaert E., Descheemaeker M.-J., Van Eylen L., Noens I., Steyaert J., Legius E. (2015). Prevalence of Autism Spectrum Disorder symptoms in children with neurofibromatosis type 1. Am. J. Med. Genet. Part B.

[109] Hoeffer C.A., Sanchez E., Hagerman R.J., Mu Y., Nguyen D.V., Wong H., Whelan A.M., Zukin R.S., Klann E., Tassone F. (2012). Altered mTOR signaling and enhanced CYFIP2 expression levels in subjects with Fragile X syndrome. Genes Brain Behav..

[110] Tsai V., Parker W.E., Orlova K.A., Baybis M., Chi A.W.S., Berg B.D., Birnbaum J.F., Estevez J., Okochi K., Sarnat H.B. (2014). Fetal Brain mTOR Signaling Activation in Tuberous Sclerosis Complex. Cereb. Cortex.

[111] Sato A., Kasai S., Kobayashi T., Takamatsu Y., Hino O., Ikeda K., Mizuguchi M. (2012). Rapamycin reverses impaired social interaction in mouse models of tuberous sclerosis complex. Nat. Commun..

[112] Butler M.G., Dasouki M.J., Zhou X., Talebizadeh Z., Brown M., Takahashi T.N., Miles J.H., Wang C.H., Stratton R., Pilarski R. (2005). Subset of individuals with autism spectrum disorders and extreme macrocephaly associated with germline PTEN tumour suppressor gene mutations. J. Med. Genet..

[113] Kwon C.H., Luikart B.W., Powell C.M., Zhou J., Matheny S.A., Zhang W., Li Y., Baker S.J., Parada L.F. (2006). Pten Regulates Neuronal Arborization and Social Interaction in Mice. Neuron.

[114] Cupolillo D., Hoxha E., Faralli A., De Luca A., Rossi F., Tempia F., Carulli D. (2015). Autistic-Like Traits and Cerebellar Dysfunction in Purkinje Cell PTEN Knock-Out Mice. Neuropsychopharmacology.

[115] Johannessen C.M., Reczek E.E., James M.F., Brems H., Legius E., Cichowski K. (2005). The NF1 tumor suppressor critically regulates TSC2 and mTOR. Proc. Natl. Acad. Sci. USA.

[116] Burket J.A., Benson A.D., Tang A.H., Deutsch S.I. (2014). Rapamycin improves sociability in the BTBR T+itpr3tf/J mouse model of autism spectrum disorders. Brain Res. Bull..

[117] Berry A., Bindocci E., Alleva E. (2012). NGF, Brain and Behavioral Plasticity. Neural Plast..

[118] Gnahn H., Hefti F., Heumann R., Schwab M.E., Thoenen H. (1983). NGF-Mediated increase of choline acetyltransferase (ChAT) in the neonatal rat forebrain: Evidence for a physiological role of NGF in the brain?. Dev. Brain Res..

[119] Li Y., Holtzman D.M., Kromer L.F., Kaplan D.R., Chua-Couzens J., Clary D.O., Knüsel B., Mobley W.C. (1995). Regulation of TrkA and ChAT expression in developing rat basal forebrain: Evidence that both exogenous and endogenous NGF regulate differentiation of cholinergic neurons. J. Neurosci..

[120] Riikonen R., Vanhala R. (1999). Levels of cerebrospinal fluid nerve-growth factor differ in infantile autism and Rett syndrome. Dev. Med. Child Neurol..

[121] Nelson K.B., Grether J.K., Croen L.A., Dambrosia J.M., Dickens B.F., Jelliffe L.L., Hansen R.L., Phillips T.M. (2001). Neuropeptides and neurotrophins in neonatal blood of children with autism or mental retardation. Ann. Neurol..

[122] Stamova B.S., Tian Y., Nordahl C.W., Shen M.D., Rogers S., Amaral D.G., Sharp F.R. (2013). Evidence for differential alternative splicing in blood of young boys with autism spectrum disorders. Mol. Autism.

[123] Chakrabarti B., Dudbridge F., Kent L., Wheelwright S., Hill-Cawthorne G., Allison C., Banerjee-Basu S., Baron-Cohen S. (2009). Genes related to sex steroids, neural growth, and social-emotional behavior are associated with autistic traits, empathy, and asperger syndrome. Autism Res..

[124] Lu A.T.-H., Yoon J., Geschwind D.H., Cantor R.M. (2013). QTL replication and targeted association highlight the nerve growth factor gene for nonverbal communication deficits in autism spectrum disorders. Mol. Psychiatry.

[125] Ellegood J., Anagnostou E., Babineau B.A., Crawley J.N., Lin L., Genestine M., DiCicco-Bloom E., Lai J.K.Y., Foster J.A., Peñagarikano O. (2015). Clustering autism: Using neuroanatomical differences in 26 mouse models to gain insight into the heterogeneity. Mol. Psychiatry.

[126] Kim H., Lim C.-S., Kaang B.-K. (2016). Neuronal mechanisms and circuits underlying repetitive behaviors in mouse models of autism spectrum disorder. Behav. Brain Funct..

